# Effect of Splinting on Orthodontic Mini-Implant Tipping and Bone Histomorphometric Parameters: An In Vivo Animal Model Study

**DOI:** 10.3390/jfb14050239

**Published:** 2023-04-24

**Authors:** Joana Fontes, Victor Zacharias Martin, Marta Resende, Bruno Colaço, Pedro de Sousa Gomes, José Manuel Amarante

**Affiliations:** 1 Faculty of Medicine, University of Porto, 4200-319 Porto, Portugal; 2 BoneLab—Laboratory for Bone Metabolism and Regeneration, Faculty of Dental Medicine, University of Porto, Rua Dr. Manuel Pereira da Silva, 4200-393 Porto, Portugal; 3 LAQV/REQUIMTE—Laboratório Associado para a Química Verde/Rede de Química e Tecnologia, University of Porto, 4100-007 Porto, Portugal; 4 Faculty of Dental Medicine, University of Porto, 4200-393 Porto, Portugal; 5 Department of Zootechnics, University of Trás-os-Montes and Alto Douro (UTAD), 5000-801 Vila Real, Portugal; 6 Center for the Research and Technology of Agro-Environmental and Biological Sciences (CITAB), University of Trás-os-Montes and Alto Douro (UTAD), 5000-801 Vila Real, Portugal; 7 Associate Laboratory for Animal and Veterinary Sciences (AL4AnimalS), 5000-801 Vila Real, Portugal

**Keywords:** mini-implants, splinting, bone tissue, tipping, bone histomorphometry

## Abstract

This study aimed to address the stability of orthodontic mini-implants submitted to an immediate orthodontic functional load, in splinted or unsplinted conditions, further characterizing the histomorphometric parameters of the neighboring bone tissue, in an *in vivo* experimental model. Mini-implants (1.4 × 6.0 mm) were placed in the proximal tibia of New Zealand White rabbits and immediately loaded with a 150 g force. Tissue healing was characterized within 8 weeks. Microtomography was used to assess the mini-implants’ tipping and bone histomorphometric indexes. Loaded implants were evaluated in splinted and unsplinted conditions, with data being compared to that of unloaded mini-implants with the Kruskal–Wallis nonparametric test, followed by Dunn’s multiple comparison tests. The splinting of mini-implants submitted to immediate orthodontic loading significantly reduced the tipping to levels similar to those of unloaded mini-implants. Immediate loading further increased the histomorphometric indexes associated with bone formation at the peri-implant region, in both splinted and unsplinted conditions, with no significant differences between the tension and compression regions. Accordingly, within this experimental setting, splinting was found to lessen tipping and mini-implants’ displacement, without affecting the increased bone formation at the peri-implant region, induced by a functional orthodontic load.

## 1. Introduction

In clinical orthodontic practice, a stable anchorage is essential to withstand the reactive forces that derive from tooth movements, further preventing the development of negative effects [1]. Traditionally, anchorage has been attained by the use of extra-oral or intra-oral appliances, with associated disadvantages—such as the elaborate design, the need for high patient compliance, and most importantly, the limited efficacy during active appliance therapy [2,3].

In order to cope with these hindrances, innovative solutions, relying on the skeletal anchorage upon the mini-implants’ placement, have become increasingly popular, allowing a paradigm shift in the expected movement of the teeth resulting from orthodontic biomechanics. Currently, many types of mini-implants are available, characterized by distinct sizes, shapes, and design characteristics. From a structural point of view, mini-implants are composed of the head—the part exposed to the oral environment, which should permit the retention of the orthodontic accessory—the transgingival component—which allows the transition between the endosseous part of the mini-implant and the head—and the threadable portion—which has a cylindrical, conical, or truncated cone design and aims at stability via direct contact with the bone tissue. Mini-implants usually range between 1.5 and 2.5 mm in diameter and between 6 and 10 mm in length, with different lengths of the transgingival element to allow a proper adaptation of the mucosa of different thicknesses. Mini-implants, given their small size, allow for a high treatment versatility, simple operation, and high efficacy, improving the effectiveness of new treatment options with solid control over the spatial tooth displacement [4,5]. Initially limited to clinical conditions in which high anchorage requirements were essential for the maximum retraction of the anterior teeth, the application to a wide range of complex three-dimensional tooth movements—including intrusion, extrusion, distalization, expansion, and protraction—has been envisioned, with a very high success rate achieved—above 80–90% [6,7]. This notwithstanding, differences in the success rates seem to occur in relation to the location of insertion, with mandibular placement presenting an increased risk of failure as compared to placement in the maxilla, as well as the option of inter-radicular placements in comparison to extra-alveolar locations, such as palatal placement [8,9,10]. Mini-implants further offer the benefits of a relatively low cost, placement in a single chairside procedure, better patient compliance, and the possibility for immediate loading, as osseointegration is not a requirement for effective functionality.

Successful clinical outcomes seem to be greatly correlated with the stability of mini-implants, broadly defined by two major components: primary stability—achieved due to the mechanical interlocking of the mini-implant within the surrounding bone during insertion—and secondary stability—proceeding from continuous bone healing or the remodeling process at the bone–implant interface and neighboring tissues [11]. Different factors seem to influence the stability and, consequently, the survival and success rate of mini-implants, broadly including patient-related (e.g., age, gender, oral hygiene levels, type of malocclusion, location of placement, soft tissue conditions, local bone quality, and clinical purpose), mini-implant-related (e.g., geometry, diameter, length, and thread characteristics), and/or technique-related (e.g., operator experience, surgical protocol, loading protocol—strength and duration of the orthodontic forces) parameters, despite the inconsistent data from recent systematic reviews and meta-analytical studies [11,12,13,14].

Furthermore, mounting evidence has converged on the idea that, upon orthodontic loading, mini-implants may not remain completely stationary, despite their overall functional stability [15]. Whether absolute anchorage—the absence of the movement of mini-implants in response to the forces applied to induce teeth movement—is envisaged [5], clinical evidence supports the displacement of mini-implants under functional orthodontic loadings and force application [16]. Although primary displacement—resulting from the elastic characteristics of the supporting bone and associated tissues—does not seem to be of clinical relevance for implant failure or mobility, given the low range of values—below to 0.1 mm [15]—significant secondary displacement under orthodontic forces—due to the remodeling processes—has been described [15,16]. This displacement may affect the integrity of distinct anatomical structures, such as dental roots and other notable structures, and further compromise the outcomes of the orthodontic treatment [15]. Recent biomechanical and experimental in vivo studies have validated the positional shift of mini-implants under functional orthodontic loading, in direct relation to the magnitude of the force, further evidencing a reduction of the displacement velocity with time [17,18,19]. Most interestingly, increased bone formation has also been attained at the bone-to-implant surface and peri-implant region [17,18].

To minimize displacement, the splinting of mini-implants has been considered. In this frame, oral rehabilitation approaches with splinted mini-implants have been successful [20,21], evidencing decreased bone stress levels and reduced marginal bone loss, in comparison to rehabilitated unsplinted mini-implants [22]. Nonetheless, few data reports on the functionality of splinted orthodontic mini-implants are available, and the studies conducted seem to be broadly limited, to the best of the authors’ knowledge, to in vitro protocols [23] or clinical case reports [24,25]. Accordingly, this study aimed to address the stability of orthodontic mini-implants submitted to an orthodontic functional load, in splinted or unsplinted conditions, placed in a translational experimental in vivo model. For this, a detailed microtomographic analysis was conducted, assessing mini-implant tipping and the distinct histomorphometric parameters of the neighboring bone tissue—further including a segmentation into zones of tension or compression—to disclose the potential influence of the forces on the biological outcomes. The null hypothesis was that there are no significant differences regarding the tipping or bone histomorphometric indexes, between splinted and unsplinted loaded orthodontic mini-implants.

## 2. Materials and Methods

### 2.1. Materials

Within the present study, the following materials were used: 60 self-tapping mini-implants (VectorTAS^TM^, Ormco, Amersfoort, The Netherlands) with a 6 mm length and a 1.4 mm diameter; 20 closed coil springs (ALIGN™ coil springs, Ormco, Amersfoort, The Netherlands); 10 splints, manufactured in a cobalt-chromium-molybdenum (Co-Cr-Mo) alloy (analytical composition of Co 61%, Cr 25%, Mo 6%, and others 8%).

### 2.2. Experimental Groups

**Figure 1:**
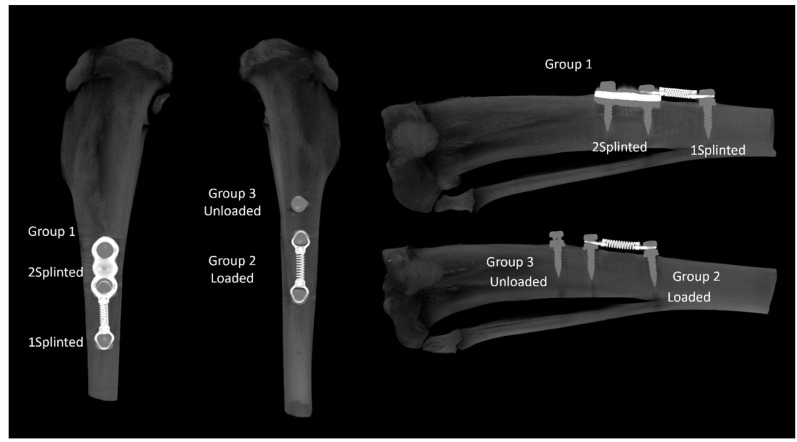
Representative microtomographic images of the proximal tibia disclosing the experimental groups. **Left**—anterior view; **right**—sagittal section.

In this work, the following experimental groups were considered (Figure 1):

- Group 1—Two splinted mini-implants, connected to another mini-implant through a coil spring. Within this group, two sub-groups were independently analyzed: splinted implants (2 Splinted) and implants connected to the coil spring (1 Splinted);
- Group 2—One mini-implant, connected to another mini-implant through a coil spring (Loaded);
- Group 3—One unloaded mini-implant (unloaded).

In order to guarantee the effectiveness of the load between the fixtures, the coil extension corresponding to a 150 g force was previously determined with a dynamometer (Correx, Haag-Streit Diagnostics, Mason, OH, USA). It was verified that a separation of 12.5 mm corresponded to the desired load, being thus-defined as the distance between the fixtures that would be submitted to the load.

### 2.3. Animals

Ten male New Zealand white rabbits, with a mean weight of 2.95 ± 0.40 kg, were acquired from a certified vendor (Granja San Bernardo, Navarra, Spain). The animals were acclimatized for 2 weeks before any experimental intervention, in order for the animals to adjust to the new environment. The animals were randomized into groups and maintained in clearly labeled individual cages with environmental enrichment. Throughout the experimental period, the animals were housed in a controlled temperature and humidity room, with a 12 h light/dark cycle and controlled ventilation. The animals had *ad libitum* access to food (2RB19, Complete feed for rabbit, Mucedola, Milano, Italy) and water.

### 2.4. Surgical Procedure

Animals were pre-medicated with diazepam at 1 mg/kg (intramuscular administration) and anesthetized with ketamine at 30 mg/kg and xylazine at 5 mg/kg (intramuscular administration), supplemented with buprenorphine at 0.03 mg/kg (subcutaneous administration), for intraoperative analgesia. Mepivacaine 3% was infiltrated within the surgical area, and fluid therapy with sterile saline was initiated and maintained throughout the surgical procedure. The eyes were treated with an ophthalmic ointment to prevent drying and corneal damage. Supplemental heat was provided during the anesthetic procedure and during recovery, to prevent hypothermia.

Upon assessment of the anesthetic plane and monitoring, the trichotomy of the anterior area of the proximal tibia was established, followed by skin disinfection with chlorhexidine 2%. The limbs were positioned for orientation, and a full-thickness incision was established followed by careful tissue blunt dissection, for mucoperiosteum elevation and exposure of the bone surface. Prior to the placement of the mini-implants, cortical drilling was conducted with a spherical drill of 0.5 mm in diameter, at a low speed. Following this, the mini-implants were placed with a surgical engine, with a torque lower than 20 Ncm. In the splinted conditions, the vertex of the implants’ heads was longitudinally aligned to ensure the proper splint position. In selected conditions (Groups 1 and 2), the spring coil was applied to the coronal portion of the mini-implants, separated by 12.5 mm, along the main axis, immediately following implantation—immediate loading. Following this, the tissues and the skin were closed in layers with 5/0 absorbable sutures (Safil, BBraun, Queluz, Portugal). Infection prophylaxis was administered using enrofloxacin 10 mg/kg.

Post-operative recovery was closely monitored, namely body temperature and food ingestion, to prevent post-operative ileus, with high-value treat food being offered as soon as possible. During the postoperative period, animals were allowed free movement in the cages, and an analgesic regimen with buprenorphine was maintained for 8 days. The animals’ situation was assessed daily regarding feeding condition, weight, body temperature, breathing, the clinical appearance of the surgical site, movement function, and signs of pain and distress.

### 2.5. Sample Collection

Animals were euthanized with an anesthetic overdose of pentobarbital upon 8 weeks of healing. To identify potential systemic alterations, a systematic necropsy was conducted with the histopathological characterization of the liver and kidneys, with a particular focus on potential clinical alterations and changes associated with the region of implantation. The tibias were subsequently separated *en bloc*, and the soft tissues were carefully removed, prior to fixation. The soft tissues surrounding the mini-implants were only removed upon a detailed gross examination.

### 2.6. Microtomographic Analysis

**Figure 2:**
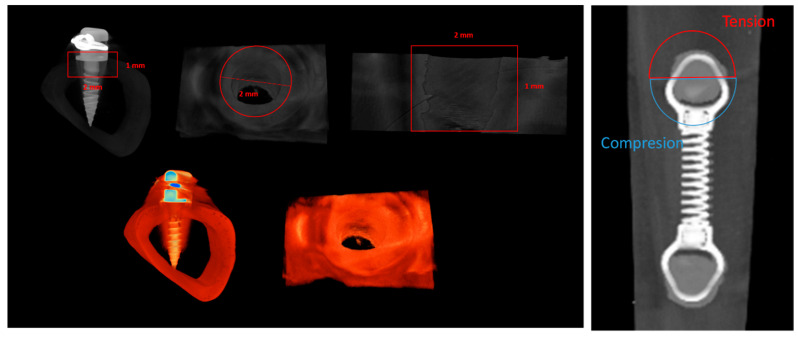
Representation of the definition of the volume of interest (VOI) for the morphometric analysis in representative microtomographic images, including all the peri-implant region (**left**) or the segmentation into tension and compression regions (**right**).

The tibias, previously fixed in 70° alcohol, were scanned using a Skyscan 1276 microtomographic system (Bruker micro CT NV, Kontig, Belgium), with 70 kV, 100 μA, and a 7.5 μm voxel size resolution. Scans were conducted with the following characteristics: rotation step of 0.2°, 360° rotation, and framing averaging. Images were reconstructed in NRecon software (Bruker, Version 1.7.4.2) with defined parameters regarding ring artifact correction [7], beam hardening correction (5%), and a minimum/maximum CS to image conversion of 0.0 to 0.16. The reconstructed data were aligned along the sagittal axis using the DataViewer software (Bruker, Version 1.5.6.3) to produce new transaxial image files, which were subsequently exported to histomorphometric analysis, performed using the CTAnalyser software (Version 1.17.7.2). Three-dimensional images were generated using the CTVol software (Brucker, Version 2.3.2.1), and representative samples were acquired with the CTVox software (Bruker, Version 3.3.0). Histomorphometric analyses of the bone tissue around the mini-implants were performed on the CTAnalyser, following established methodologies [26,27,28]. The volume of interest (VOI) for the morphometric analysis was defined as a cylinder of 2 mm in diameter and 1 mm in height, centered on each implant (Figure 2, left). Images were segmented with 2 distinct thresholds, varying the lower and upper grey limits, for the definition of the densities of the implant and bone. Morphometric analyses were conducted to determine the total volume (TV)—the integral volume of the VOI, in mm^3^—bone volume (BV)—the volume of the VOI segmented as bone tissue, in mm^3^—and bone surface (BS)—the surface of the VOI segmented as bone tissue, in mm^2^. The defined parameters allowed the determination of the following indexes: bone volume fraction (BV/TV), defined as the ratio of the segmented bone volume to the total volume of the VOI; the specific bone surface (BS/BV), defined as the ratio of the segmented bone surface to the segmented bone volume of the VOI; the bone surface density (BS/TV), defined as the ratio of the segmented bone surface to the total volume of the VOI; and the bone mineral density (BMD), defined as the coefficient of the linear attenuation converted for the physical density of hydroxyapatite, in g.cm^3^ of hydroxyapatite, calibrated using an appropriate phantom. The angulation of the tip (tipping) was further determined by the deviation from the perpendicular plan, at the implant insertion location. In selected conditions, independent analyses were conducted on the hemicylinders defined as new VOIs, delimitating the regions submitted to “compression” or “tension” forces, in accordance with the representation in Figure 2, right. All the used software were acquired from a Bruker microCT NV, Kontig, Belgium.

### 2.7. Statistical Analysis

Statistical assessment was conducted with the SPSS software (SPSS Statistics 27, Chicago, IL, USA). In the quantitative analysis, the data are presented as the mean ± standard deviation (SD), considering the different replicates of the same experimental condition. The variables were compared using the Kruskal–Wallis nonparametric test, followed by Dunn’s multiple comparison tests, with *p* ≤ 0.05.

### 2.8. Ethical Issues

All husbandry, protocols, and procedures involving the maintenance and use of the animals in the frame of this study were reviewed and approved by the national competent authority (Food and Veterinary Directorate-General (DGAV)), under Project License No. 010532/2018. All procedures were further performed under the European Directive 2010/63/EU and the National Law (DL No. 113/2013), considering possible replacement, reduction, and refinement strategies. All procedures were conducted by certified researchers for the practice with animals used for scientific purposes. The manuscript was prepared according to the ARRIVE guidelines.

## 3. Results and Discussion

In the present study, the stability of functionally loaded orthodontic mini-implants was evaluated in splinted and unsplinted conditions, by the microtomographic assessment of tipping and distinct histomorphometric parameters within the neighboring bone tissue. Given the data obtained, the null hypothesis that there is no significant difference in mini-implant tipping and histomorphometric bone parameters, in splinted and unsplinted orthodontic mini-implants submitted to a functional load, was rejected.

Overall, the postoperative period was uneventful for all animals, with no reported signs of infection, ulceration, altered tissue structure or organization at the surgical wound, or acknowledged adverse effects. All animals gained weight between the preoperative day and the end of the experimental period—upon 8 weeks of healing. At the necropsy, no significant clinical alterations or histopathological alterations were systemically noticed, nor adverse tissue responses at the implantation area—in line with the verified functional activity of all the placed mini-implants.

The primary stability of mini-implants, in the absence of osseointegration, seems to derive from the mechanical interlocking with the subjacent bone tissue—substantiating the importance of bone quality and quantity within the implantation region [5]. Accordingly, the rabbit’s proximal tibia was selected as a suitable anatomical location for the implantation of the complex experimental splint design, allowing translational assessment of the morphological and functional characteristics of the oral bone response [29]. The rabbits’ proximal tibia offers a standardized and easily accessible anatomical location, with a thick cortical structure in which new bone formation derives from the endosteum, being regarded as an adequate model for implant-related research [30]. This notwithstanding, translational analyses must not disregard the distinctive morphological arrangement, composition, and remodeling kinetics of rabbits’ bones, as compared to those of humans [30].

**Figure 3:**
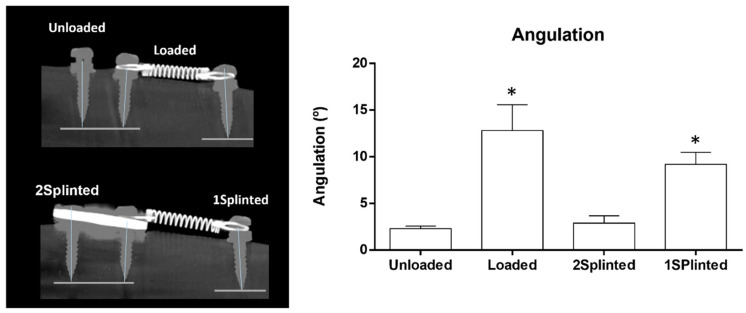
Representative microtomographic images of the tipping analysis (**left**) and quantitative histomorphometric data on tipping angulation (**right**). * Significantly different from the Unloaded condition (*p* ≤ 0.05).

The explanted tibias were subsequently characterized and analyzed by microtomography regarding distinct histomorphometric parameters, according to the defined experimental groups. The tipping of the mini-implants was also evaluated and quantified (Figure 3). Briefly, a low deviation from the perpendicular was verified both in the Unloaded and 2 Splinted conditions, while a higher deviation was attained in the 1 Splinted and Loaded conditions, as verified in the microtomographic analysis (Figure 3, left). The quantitative assessment revealed a low angulation—lower than 3° in both the Unloaded and 2 Splinted groups—while a significantly higher tipping value (over 10°, *p* ≤ 0.05) was verified in the 1 Splinted and Loaded conditions (Figure 3, right). Overall, regarding the mini-implants submitted to the orthodontic load, microtomographic data evidenced that splinting was effective in lessening tipping. The splinted mini-implants presented tipping levels similar to those of unloaded implants and significantly less than those of unsplinted mini-implants. Previous studies reported that, despite providing good anchorage quality, unsplinted mini-implants are subjected to both primary and secondary displacement, which may reach the millimeter range in diverse directions [15]. Controlled tipping seems to be the most-common type of movement, ranging from mean values of around 2 mm in the same direction to −1 mm in the opposite direction of the established orthodontic force [31,32]. On the other hand, whole mini-implant displacement seems to reach mean levels of about 2.7 mm, with maximal values up to 5.5 mm [33,34]. Displacement seems to further relate to the intensity of the orthodontic loading—low forces broadly induce tipping, while high forces also induce displacement at the mini-implant head, with further alterations in the velocity of migration with time [18]. From a biological point of view, the immediate orthodontic loading may preclude the integration of the surrounding tissues with the implant surface, leading to localized resorption—as a mechanical reaction, given the viscoelastic properties of the bone [35]. Splinting is expected to augment the functional surface area accountable for the interaction and support by peri-implant tissues, improving stability and allowing for an improved load distribution [36]. This could possibly reduce the focalization of the initial peri-implant tissue strain, at the bone–implant interface, providing a decreased mini-implant displacement and/or tipping upon functional loading [37].

Splinting may, thus, be regarded as a mechanical upgrade, able to increase the therapeutic versatility of mini-implants in orthodontic therapeutic approaches, in particular in clinical situations in which tipping and mini-implant displacement may be prejudicial to the neighboring anatomical structures or significantly influence the treatment outcomes. By increasing the area of anchorage with the splinting apparatus, the interaction with the teeth’s center of resistance may be modulated, allowing modifications and fine-tuning of the mode and direction elements of the tooth movement, further improving the clinical outcomes [38]. On the other hand, splinting requires the placement of multiple mini-implants, which widens the surgical area and the soft and hard tissue healing requirements, potentially increasing the risk of trauma or tissue damage. Complementary techniques, such as digital treatment planning and surgical guides, may be used to overcome these hindrances and facilitate the clinical implementation of splinting in orthodontic applications [39,40].

**Figure 4:**
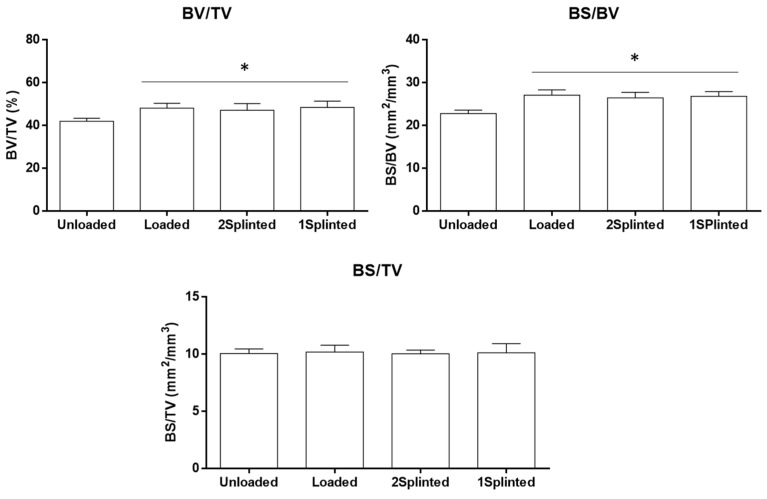
Histomorphometric parameters (BV/TV—bone volume fraction; BS/BV—specific bone surface; BS/TV—bone surface density) of the microtomographic analysis at the defined VOI. * Significantly different from the Unloaded condition (*p* ≤ 0.05).

Despite the acknowledged displacement of mini-implants upon immediate orthodontic loading, the bone formation process does not seem to be hindered at the bone–implant interface [41]. In order to disclose potential differences in the tissue response to both splinted and non-splinted mini-implants, the bone histomorphometric parameters of the region neighboring the placed constructs were determined, based on microtomographic datasets (Figure 4). Microtomography has become a methodology of relevance for the analysis of the three-dimensional morphology and architecture of the bone-to-implant interface and neighboring bone parameters, exceeding the limitations associated with the methodological preparation of samples for histological analysis and restricted bidimensional assessment of the structures [42,43]. Briefly, the BV/TV was found to be high on the assessed VOIs. Comparatively, implants from the 2 Splinted, 1 Splinted, and Loaded groups presented significantly higher levels than those of the Unloaded group (*p* ≤ 0.05), with no significant differences between loaded conditions. A similar trend was attained for the BS/BV, with all experimental groups presenting significantly higher levels than that of the Unloaded condition (*p* ≤ 0.05), with no significant differences between conditions submitted to a functional orthodontic load. Regarding the BS/TV, no significant differences were verified between the conditions. The BV/TV is regarded as the main parameter to evaluate the bone quantity, reflecting the proportion of mineralized tissue within the volume of interest. The BS/BV and BS/TV are structural parameters disclosing the measure for the bone surface per given bone volume, or tissue volume, respectively, being valuable for the characterization of the complexity of the bone structure [44]. Comparatively, increased BV/TV levels suggest increased bone formation, while increased BS/BV levels reflect an increased active surface area per unit of bone, correlating with an increase in bone remodeling, at a specific site [45].

Previous studies have suggested that immediate orthodontic loading does not hamper the success rate of mini-implants within experimental in vivo assays [46] and clinical trials [47]. In fact, despite the acknowledged tipping, as presently verified, orthodontic loading seems to modulate the structure and properties of the bone tissue at the peri-implant region. In distinct experimental models, an increased bone remodeling associated with an increased deposition rate has been verified in functional orthodontic loading conditions [48,49,50]. Functional loading has been found to increase the turnover from immature to mature bone tissue, increasing the velocity of lamellar bone production [48]. The increased remodeling might further contribute to the prevention of microdamage and crack accumulation at the interfacial bone–implant tissue, increasing the overall peri-implant bone properties [51].

**Figure 5:**
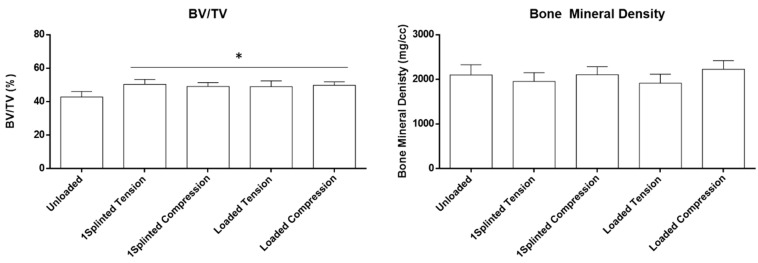
Histomorphometric parameters (BV/TV—bone volume fraction; bone mineral density) of the microtomographic analysis of the VOI, segmented for compression and tension regions. * Significantly different from the Unloaded condition (*p* ≤ 0.05).

In order to disclose potential dissimilar influences of tension or compression forces on the biological outcomes, the defined VOI around the mini-implants was segmented into volumes submitted to tension or compression forces (Figure 2, right), in which the BV/TV and bone mineral density were determined (Figure 5). Briefly, loaded mini-implants—either splinted or unsplinted—presented significantly higher levels of the BV/TV in either tension or compression regions, than those attained for the unloaded control (*p* ≤ 0.05). In addition, no significant differences were disclosed between the experimental conditions. In regard to the bone mineral density assessment, no significant differences were verified between the experimental conditions and control, regardless of the region being submitted to tension or compression forces. Overall, the assessment of the segmented datasets supports increased bone formation upon loading, regardless of the established stresses at the interfacial region, with a similar degree of mineralization to the unloaded control. This sustains a physiological distribution of the mineral content within the increased newly formed bone volume, in response to functional orthodontic loading.

Bone formation at the peri-implant region submitted to functional and constant orthodontic loading, in regions of tension or compression, remains a controversial topic in the literature. Some works have reported significant differences in bone deposition, morphometric parameters, and/or biomechanical properties, between regions submitted to dissimilar forces [52,53]. Broadly, a trend for an increased remodeling on the compression side has been determined when the differences were reported between the two sides [54,55] and attributed to a specific range of the applied force magnitude and biological response of the deformed bone [56]. More recent reports, in line with the data obtained in this study, identified no significant differences in the bone parameters, between tension or compression regions submitted to a functional orthodontic load [57,58]. It is possible that the obtained differences may relate to variations within the experimental design, including dissimilarities in the selected animal model, the characteristics of the used mini-implants, the force and loading protocols, the location of implantation, the period of evaluation, the methodology, and the characterization methodologies, among other variables. Regardless of the outcomes, it has been recently suggested that the stress induction at the mini-implant interface—in spite of the prevalence of tension or compression forces—may be the trigger for bone remodeling, further enhancing the appositional process, leading to enhanced bone formation [18]. At the molecular level, whether no significant differences were obtained regarding the osteocytic gene expression program, variations in the expression of CTSK—coding for cathepsin k, a protease acknowledged as a major marker of the osteoclastic activity [59]—and those of RUNX2 and SP7—coding for Runt-related transcription factor 2 and Osterix, respectively, two major transcription factors regulating the osteogenic program [59]—were obtained in a site- and time-dependent manner [18]. Histological analysis [18] further evidenced the presence of active cells—osteoclasts, osteoblasts, and respective precursors—supporting the increased remodeling, in close association with increased angiogenesis—the formation of vascular structures from pre-existing ones through sprouting—a recognized vital process for bone homeostasis, in both remodeling and healing activities [60].

This trend is supported by integrative studies, combining finite-element analysis with biological data, which have showcased increased bone formation in regions submitted to a higher strain intensity in both mini-implants [61] and natural teeth [62], in response to a functional load. Comparatively, mini-implants seem to allow a higher concentration of maximal stress responses upon loading, in relation to natural teeth. The periodontal ligament—exclusively associated with natural teeth—is expected to contribute to the absorption and dissipation of the mechanical stresses, given the acknowledged viscoelastic properties. The structural rigidity and stiffness of the mini-implants may further contribute to the increased stress levels at the bone interface [63] and, expectedly—within a selected range—contribute to an enhanced bone formation outcome [64]. In this regard, active bone cells—including both osteoblasts and osteoclasts, as well as their respective precursors—have been shown to activate distinct mechanical transduction pathways that are known to modulate cellular behavior, including proliferation, differentiation, and functional activity, in response to distinct forms of mechanical stress of the local microenvironment, in which both compressive and tensile stresses are included [65]. Of additional relevance, the induction of fluid shear stress—by the flow of tissue fluid at the lacunar-canalicular bone system [66]—has recently been found to modulate the mechanical transduction of the osteoblastic behavior at the bone-to-implant interface, via the enhancement of the cell proliferation and differentiation [67], potentially contributing to the attained outcomes.

Even if the obtained data have been observed upon 8 weeks of healing in a translational rabbit model, this may not disclose the biological differences regarding the bone structure and the metabolism of bone healing between humans and rabbits, nor the need to address the biological evaluation for longer time periods, in order to translate the long-term efficacy of splinting into the clinical orthodontic setting.

Splinting may, thus, be regarded as a mechanical upgrade, able to increase the therapeutic versatility of mini-implants in orthodontic therapeutic approaches, in particular in clinical situations in which tipping and mini-implant displacement may be prejudicial to the neighboring anatomical structures or significantly influence the treatment outcomes. By increasing the area of anchorage with the splinting apparatus, the interaction with the teeth’s center of resistance may be modulated, allowing modifications and fine-tuning of the mode and direction elements of the tooth movement, further improving the clinical outcomes. On the other hand, splinting requires the placement of multiple mini-implants, which increases the surgical area, soft and hard tissue healing requirements, and potentially, the risk of trauma or tissue damage. Complementary techniques, such as digital treatment planning and surgical guides, may be used to overcome these hindrances and facilitate the clinical implementation of mini-implant splinting in orthodontics.

## 4. Conclusions

Overall, splinting of mini-implants submitted to orthodontic functional loading was found to significantly reduce tipping to values similar to those attained in unloaded mini-implants, in a rabbit experimental model. Additionally, immediate loading was found to increase the bone volume fraction in either splinted or unsplinted mini-implants, with no significant differences between the tension and compression regions of the neighboring bone tissue. This study, to the best of the authors’ knowledge, is the first to address the mini-implant displacement and bone morphometric indexes in splinted mini-implants submitted to orthodontic loading. In general, splinting is, thus, expected to lessen tipping, as well as the displacement of mini-implants, without affecting the biological events at the peri-implant tissues—characterized by increased bone formation—acknowledged to be induced by a functional orthodontic load.

## Data Availability

The data that support the findings of this study are available upon request from the corresponding author.
